# Transcorporal artificial urinary sphincter in radiated and non - radiated compromised urethra. Assessment with a minimum 2 year follow-up

**DOI:** 10.1590/S1677-5538.IBJU.2015.0329

**Published:** 2016

**Authors:** Erwann Le Long, John David Rebibo, Francois Xavier Nouhaud, Philippe Grise

**Affiliations:** 1 Department of Urology-Rouen University Hospital-Charles Nicolle, Rouen, France

**Keywords:** Suburethral Slings, Urinary Incontinence, Urogenital System, Radiation

## Abstract

**Purpose:**

to assess the efficacy of transcorporal artificial urinary sphincter (AUS) implantation on continence for male stress urinary incontinence in cases of prior surgical treatment or/and radiation failure, and as a first option in radiation patients.

**Materials and Methods:**

From March 2007 to August 2012, 37 male patients were treated with transcorporal AUS AMS™ 800. Twelve patients had primary placement of transcorporal cuff, a surgical option due to a previous history of radiation and 25 patients had secondary procedure after failure of AUS or urinary incontinence surgery. Functional urinary outcomes were assessed by daily pad use, 24-hour Pad-test and ICIQ-SF questionnaire. Quality of life and satisfaction were assessed based on I-QoL and PGI-I questionnaires.

**Results:**

After a median of 32 months, the continence rate (0 to 1 pad) was 69.7%. Median pad test was 17.5g (0-159), mean ICIQ-SF score was 7.3/21 (±5.4) and mean I-QoL score was 93.9/110. A total of 88% of the patients reported satisfaction with the AUS. The 5-year actuarial revision-free for AUS total device was 51%. Patients for primary implant for radiation were not more likely to experience revision than non-radiation patients. Preservation of erections was reported in half of the potent patients.

**Conclusions:**

Transcorporal AUS cuff placement is a useful alternative procedure option for severe male UI treatment, especially in patients with a compromised urethra after prior surgery or radiation. A high continence rate was reported and implantation as first option in radiation patients should be considered.

## INTRODUCTION

Urinary incontinence (UI) is a serious adverse effect due to radical prostatectomy (1). After failure of conservative treatment, an artificial urinary sphincter (AUS) is considered the gold standard treatment for mild to severe incontinence from intrinsic sphincter deficiency (2). It provides a high continence rate and patient satisfaction, despite a reoperation rate that may reach 62.7% (3). In men the AUS cuff is implanted directly around the bulbar urethra. However, cuff placement may be difficult in a compromised urethra due to prior AUS placement, radiation or urethral surgery leading to a high risk of failure (4).

The transcorporal approach to AUS placement was created to protect the urethral wall in AUS revision for urethral atrophy and erosion (5) that protects the posterior wall of urethra during dissection, which may be critical.

AUS implantation using a transcorporal cuff was recently reported by Wiedemann et al. (6) that concluded that transcorporal AUS cuff placement is a useful alternative for challenging cases of male UI after failure of previous surgical treatment, urethral atrophy or erosion. The aim of the study was to conduct a selective evaluation in radiation and non-radiation patients to assess transcorporal AUS as a first option in radiation patients.

## MATERIALS AND METHODS

Patient selection: From March 2006 to August 2012, 44 patients underwent AUS (AMS800®) implantation with a transcorporal cuff by the same surgeon in a single center. All patients were contacted by letter and telephone for evaluation, and were examined by an independent urologist. Later all patients completed a subjective satisfaction questionnaire.

Degree of continence was assessed by a 24 hour Pad-test, validated questionnaire (International Consultation on Incontinence Questionnaire-Short Form: ICIQ-SF) and number of pads per day. Total continence was defined as no urinary leakage and no pad, social continence as 0 to 1 pad with urinary leakage. In the other cases, patients were considered as incontinent. In addition, quality of life and satisfaction were assessed based on two validated questionnaires in 33 patients who completed an Incontinence Quality of Life scale (I-QoL) and Patient Global Impression of Improvement (PGI-I) respectively.

Early postoperative complication (<30 days) was recorded. Revision was defined as any additional procedure on the AUS, including explantation of the device with or without de novo implantation at the same time. In order to assess the results and complications with a sufficient period of follow-up, all patients included in the study had a minimum of 2 years of follow-up.

This study was not submitted to the local Ethics Committee for approval because it was a retrospective assessment of clinical practice.

### Surgical procedure

The transcorporal cuff placement has been previously described (5, 6). Reservoir pressures were initially 61 to 70cm/H_2_O in all of 37 patients. Cuff sizes were 4, 4.5; 5, 5.5, 6 and 6.5cm respectively in 1, 7, 10, 15, 2 and 2 patients. All sphincters were deactivated for 6 weeks after surgery.

### Statistical analysis

Continuous data were analyzed with the nonparametric Mann-Whitney test. For categorical variables, Fisher’s exact test or Chi-Square test was used. Revision-free survival of the AUS and the cuff curves were calculated using the Kaplan-Meyer method, and the significance of differences was determined using the log-rank test. Cox multivariate regression model was used to assess the relative importance of previous radiotherapy and failure of incontinence surgery on the results. Statistical significance was defined as p<0.05 for all analyses.

## RESULTS

Patient characteristics are summarized in Table-1. Mean age at sphincter insertion was 70.1 years (±7.1).

**Table 1:** Preoperative characteristics of the study population. Some patients had more than one previous surgery.

Variable	
Mean age at AUS implantation (SD)	70.1 (±7.1)
BMI (SD)	27.2 (±3.41)
No. androgen deprivation therapy	10
No. Radical prostatectomy	34
No. Previous pelvic radiotherapy	23
No. Transurethral prostatic resection	6
No. Bladder neck contracture surgery	12
No. Urethrotomy	5
No. Urethroplasty	1
**Previous surgical treatment of UI**	
AUS	17
Bulbourethral sling	7
Balloon	1

**BMI** = body mass index

Follow-up data were available on 37 of the 44 patients. Five patients died since AUS implantation and 2 were lost to follow-up and excluded from the analysis. Median follow-up was 32 months [24-51].

Twelve patients had primary placement of transcorporal cuff because of previous radiation. For 25 patients the transcorporal cuff implantation was done in the secondary procedure after failure of UI surgery (7 male slings, 17 AUS, 1 Pro-ACTTM balloon). The total AUS device was implanted in 31 patients and the cuff alone in 6 patients with the other components of AUS left in place. Periurethral adhesions were considered for transcorporal implantation.

Mean preoperative daily pad use was 4.5 (±2.1) and 2 patients used a penile sheath. Median preoperative pad test was 530g (400-690).

Continence results were reported at the latest follow-up, median value was 32 months (24-51). The AUS remained functioning in 33 patients whereas it was explanted in 3 patients and never activated in 1 patient due to neuropathic chronic scrotal pain. The continence rate for social continence and total continence was respectively 69.7% and 12.1%. Six patients (18.2%) required more than 1 pad daily and were considered incontinent. Median pad test was 17.5g (0-159) and mean ICIQ-SF score was 7.3/21 (±5.4).

## REVISION AND COMPLICATION

AUS overall complications occurred in 18 patients (48.7%) (Table-2). Seventeen patients (45.9%) had undergone one or more surgical revisions including 7 transcorporal cuff replacements. Median time to revision was 8 months [4-16].

**Table 2:** Complications occurred during follow-up. Reported number was 31 in 18 patients.

Cause of complication	No. Pts.	Delay (months)
**Infection**	**6**	
Cuff	1	5
Balloon	2	11/13
Pump and balloon	1	0.7
All of AUS	2	3/27/3
Erosion	3	0.7/1.5/7
Urethral atrophy	2	10/27
**Mechanical failure**	**11**	
Cuff	4	3/6/7/13
Balloon	5	6/8/10/20/30
Pump	1	18
Cuff and pump	1	40
**Other**	**9**	
Scrotal hematoma	2	0.1/1.5
Balloon migration	2	10/32
Acute urinary retention	5	0.1

Mean I-QoL score was 93.9 (±15.0) and the mean post-operative PGI-I score was 1.5 (±0.8). Kaplan-Meier curve demonstrated a 5-year actuarial revision-free AUS survival of 51.0%. The history of pelvic radiation or failure of urinary incontinence surgery showed no statistically significant differences concerning continence, pad test, PGI-I, ICIQ-SF and I-QoL score.

**Figure 1:**
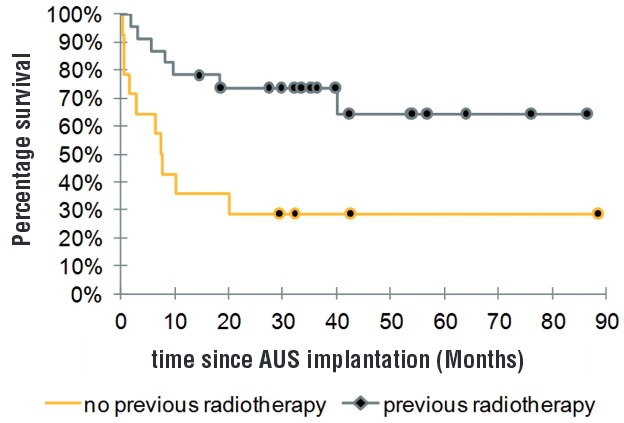
Kaplan-Meier analysis comparing the revision-free AUS survival between patients with a previous history of radiotherapy (N=23) and without (N=14) a previous history of radiotherapy. (XL STAT Microsoft Excel® Addinsoft).

Revision-free AUS survival was significantly higher in patients with previous radiation (p=0.006) (Figure-1).

In multivariate analysis, only patients with previous radiation had significantly less AUS revision than non-radiation patients (HR: 0.276; CI 95%: 0.1043-0.7295; p=0.0094).

Evaluation of outcome on potency was performed in 12 patients who presented erections before surgery, out of them 6 reported preservation of erections after the cuff implantation.

## DISCUSSION

This study represents a large number of patients with a compromised urethra, and to our knowledge the first to specifically evaluate transcorporal AUS as a first option in radiation patients.

AUS is considered the gold-standard for the treatment of moderate to severe post prostatectomy UI (7). Despite a high satisfaction rate and few complications, revisions are still required due to erosion, infection, mechanical dysfunction or sub-urethral atrophy. A previous implantation or urethral surgery or radiation had an increased risk of AUS failure.

Different management may be proposed in a compromised urethra including male sling ProACT™ and AUS (7). In cases of AUS failure due to erosion or infection, or in cases of incontinence recurrence due to sub-cuff atrophy, a tandem cuff implantation (8) has been proposed, or a relocation of the cuff to a more proximal site on the bulbar urethra (9) whether or not associated with a down-sizing of the cuff (10) by 3.5cm.

In the literature, reimplants of bulbar urethral AUS after removal of previous AUS were not associated with a lower continence rate or complication as reported by Raj et al. (9). Conversely, Lai et al. (11) found a fourfold higher risk of erosion in a secondary reimplant patients in comparison with a virgin AUS implant. In a recent study, Bant et al. (12) reported a multicenter outcome analysis of 386 AUS placement and confirmed that urethral risk factors were prior to AUS erosion, history of urethral stent placement or radiation. Mc Geady et al. (4) assessed the compromised urethra in 86 patients and found an increased rate of failure with the tandem and 3.5 cuffs. These authors observed a 67% failure rate in the 3.5 cuff group but 36% in transcorporal group. These complications of a narrow cuff were confirmed by Bant et al. (12) and could be explained by the placement in a more distal location where the urethral wall is thin and the diameter reduced.

These findings support the transcorporal AUS placement approach in a compromised urethra patient, an option which was first proposed in 2002 (5). Transcorporal AUS in salvage of AUS or sling failure occurred in 65% of our series. The various series of this technique reported in the literature are of small sample size and only one prospective study is currently available (6). In the literature, the main indication for transcorporal AUS was urethral atrophy, salvage AUS or sling failure and previous urethral surgery (5, 13-16).

The primary objective of our study was the functional results and complications evaluation. Our data were similar to those mentioned in other reported studies (5, 6, 13-16). It is difficult to compare the continence rate due to absence of a standardize definition of continence. Based on a more common definition of continence (0 to 1 pad), our continence rate was 81% and most patients were very satisfied. The complete continence rate was only 12% but this rate is difficult to assess from the literature. Our revision rate was higher than that reported in the literature but this was primarily due to mechanical failure (29.7%). This is possibly explained by an option to replace a standard placement cuff by a transcorporal cuff when the balloon and the pump were considered correct, instead of a total component replacement. There is a limited incision and a shorter operative time benefit from this option but there is also an increased the risk of secondary AUS revision for the components not changed. Another option could have been to routinely change all the components when the cuff is affected, particularly after few years follow-up. We also changed the balloon from 61-70 to 71-80 in 5 patients in order to pressurize when there was a secondary leakage and low urethral pressure under the cuff. A primary implantation of 71-80mL balloon could be considered depending on the low risk of erosion in the transcorporal cuff and to improve the total continence rate but this option has not yet been evaluated. Transcorporal cuff (TC) placement is not completely protected from erosion and we report an 8% rate. An 11-24% rate was reported by Brandt et al. (12) in a non-specific large number of 119 TC placements out of 386 AUS implantation. Wiedemann et al. (6) did not report erosion in a specific series of 23 TC placement but reported a 13% infection rate that may be an associated complication. Although the cavernosum is open, the hemorrhage complications were rarely reported, possibly due to non functional erectile tissue in the majority of patients.

Radiotherapy induces ischemia and urinary late-effect radiotoxicity may induce detrusor dysfunction, abnormal compliance, over activity and urethral damage such as fibrosis or atrophy (17-19). However, some patients could have had a normal urethral appearance during surgery or limited periurethral adhesions. In patients with a bulbar urethral cuff in primary implantation, results in the literature were contradictory. For Perez et al. (17) and Gohma et al. (19), there was no difference concerning continence or revision rates between patients with or without previous radiation therapy. Raj et al. (9-20) initially reported no difference. However, one year later in a study examining risk factors, the same institution found that a prior radiation therapy and a prior explant to increased the risk of subsequent erosion. In other series, patients with a history of radiotherapy presented more revision (18, 21, 22), or infection (18), but with no impact on continence. In the literature, no study, to our knowledge analyzed the impact of radiotherapy on functional results in patients with a transcorporal cuff.

In our series, a major finding was that there was no increase in the incontinence rate in patients who received radiotherapy and surprisingly significantly less revision. Moreover, when radiation patients had a transcorporal cuff as a first option (no prior incontinence surgery) the good results persisted in a multivariate analysis. A possible explanation could include the sample size and the effect of radiotherapy on cavernous tissue that can receive up to 44 Gy units (23) but induce a fibrosis which could increase the resistance of cavernous corpus. Our rate of erosion or infection was low, i.e. 13%, and no secondary urethral atrophy occurred in radiation patients within the limit of follow-up.

An evaluation of erectile function was not the primary aim of our study. However, the risk of impotence may be considered and the patient must be informed. Wiedemann et al. (6) showed a preservation of erectile function in 5/6 patients after transcorporal AUS placement. In our study, 50% of the 12 normal erection patients had persistent erection after surgery, confirming that transcorporal cuff was not inconsistent with erections, although the IIEF5-SF questionnaire was not used. An explanation for the preservation of erection could be the location of the cuff close to the tunica albuginea, thus leaving apart the erectile tissue. Patients must be informed that implantation of an erectile prosthesis inside the corpus cavernosum is not recommended when transcorporal cuff is performed.

The limits of our study were a retrospective analysis with a limited number of patients and single center experience, as well as the lack of a pad test and preoperative questionnaire. All of the radiation patients had external beam radiation, however we did not evaluate the relationship with the dose or brachytherapy.

The interesting results of transcorporal AUS as primary option in radiation patients must be considered, and could be more documented in a prospective study in comparison with periurethral standard cuff AUS and a longer follow-up.

## CONCLUSIONS

Transcorporal AUS cuff placement is a useful alternative procedure option for the treatment of severe male UI, especially in patients with a compromised urethra after prior surgery or radiation. A high continence rate was reported and preservation of erection occurred in 50% of the patients.
